# Image-Based Localization Aided Indoor Pedestrian Trajectory Estimation Using Smartphones

**DOI:** 10.3390/s18010258

**Published:** 2018-01-17

**Authors:** Yan Zhou, Xianwei Zheng, Ruizhi Chen, Hanjiang Xiong, Sheng Guo

**Affiliations:** 1 State Key Laboratory of Information Engineering in Surveying, Mapping and Remote Sensing (LIESMARS), Wuhan University, Wuhan 430079, China; zhouyan9103@whu.edu.cn (Y.Z.); xionghanjiang@whu.edu.cn (H.X.); guogis@126.com (S.G.); 2 Collaborative Innovation Center of Geospatial Technology, Wuhan University, Wuhan 430079, China

**Keywords:** image-based localization, PDR, low-cost indoor localization, SfM

## Abstract

Accurately determining pedestrian location in indoor environments using consumer smartphones is a significant step in the development of ubiquitous localization services. Many different map-matching methods have been combined with pedestrian dead reckoning (PDR) to achieve low-cost and bias-free pedestrian tracking. However, this works only in areas with dense map constraints and the error accumulates in open areas. In order to achieve reliable localization without map constraints, an improved image-based localization aided pedestrian trajectory estimation method is proposed in this paper. The image-based localization recovers the pose of the camera from the 2D-3D correspondences between the 2D image positions and the 3D points of the scene model, previously reconstructed by a structure-from-motion (SfM) pipeline. This enables us to determine the initial location and eliminate the accumulative error of PDR when an image is successfully registered. However, the image is not always registered since the traditional 2D-to-3D matching rejects more and more correct matches when the scene becomes large. We thus adopt a robust image registration strategy that recovers initially unregistered images by integrating 3D-to-2D search. In the process, the visibility and co-visibility information is adopted to improve the efficiency when searching for the correspondences from both sides. The performance of the proposed method was evaluated through several experiments and the results demonstrate that it can offer highly acceptable pedestrian localization results in long-term tracking, with an error of only 0.56 m, without the need for dedicated infrastructures.

## 1. Introduction

Determining the pedestrian location in indoor environments using consumer smart phones has been a fundamental requirement in many applications such as path finding, emergency planning and augmented reality. Since GPS signals cannot achieve satisfactory results in indoor environments, many alternatives have been proposed. The prominent methods for the most current indoor localization technologies are based on dedicated infrastructures, such as Wi-Fi access points [1,2], Bluetooth [3,4], ultrasonic networks [5], ultra-wideband (UWB) [6] and magnetic fields [7]. However, these methods are expensive and label-extensive for large-scale deployment and suffer from discontinuous tracking during pedestrian movement [8]. Google Tango has devised a depth camera equipped smartphone that can localize itself as well as simultaneously reconstructing the indoor model [9]. However, this is more of a model reconstruction technology than a localization strategy, for the reason that keeping the camera on during walking does not conform to the common human waking mode and localizing the smartphone and simultaneously reconstructing the scene becomes computationally expensive and memory-infeasible for larger scenes. Fortunately, vision-based localization can provide visual gyroscope and visual odometer in GPS-challenging indoor spaces [10] and serve as a low-cost and high-accuracy solution in ubiquitous indoor localization.

Vision-based localization has two main approaches, which are the simultaneous localization and mapping (SLAM) approaches such as Google Tango and image-based localization. Compared with SLAM [11], reconstructing the scene model in advance and opening the camera for localization only when lost is a more appropriate approach in indoor pedestrian localization [12]. The image-based localization result can be directly treated as the pedestrian location because people tend to carry their smartphone close to their body. Given the 2D image features and the 3D scene features, the camera pose can be estimated from the 2D-3D correspondences by applying an *n*-point pose solver inside a random sample consensus (RANSAC) loop [13]. Recent affordable or free structure-from-motion (SfM) software, such as Bundler [14], VisualSfM [15] and Photoscan [16], have allowed us to reconstruct indoor scenes and thus make it possible to undertake image-based localization in indoor environments. When combined with pedestrian dead reckoning (PDR) that estimates the distance and heading measurements of every step from the accelerometer and gyroscope embedded in the smartphone [17], discrete image-based localization can be interpolated to recover a continuous pedestrian trajectory. On the other hand, the relative positioning and the error accumulation of PDR can be remedied by the high-accuracy image localization result, by providing the initial position and regular correction when drifting. Therefore, with the 3D scene model provided, combining image-based localization and PDR can complement each other and achieve self-dependent and high-accuracy localization using only smart phones, without any extra equipment.

Image-based localization was initially formulated as an image retrieval problem focused on matching a query image to an image database with geolocations [18]. When combined with the bag-of-visual-words model [19], an image retrieval system is applicable to scalable scenes from the street-level [20], to the city-level [21] and to the worldwide-level [22]. Since the image database may contain thousands of millions of images, to efficiently retrieve and localize the query images, Li et al. [23] used an iconic scene graph to create a compact summary of the global images. Chen et al. [24], on the other hand, improved the system’s robustness to perspective views and hence the recall rate, by fusing the orthogonal and perspective street images to build synthetic views. Other improvements have focused on avoiding mismatches by dealing with repetitive scenes [25] and confusing scenes [26]. Compared to our method, the image retrieval strategy can only yield coarse location estimation. Furthermore, the raw images in the database are stored independently, with ignoring the underlying geometry [27].

In contrast to the pure image retrieval approach, SfM-based localization can obtain accurate pose estimation with exact orientation and position by correlating 2D features in a query image with 3D scene features in the model. Moreover, the SfM model presents a precise summary of the scene, with each 3D point triangulated from a trace of matched features and the noisy ones eliminated and not used for the matching. Consequently, it can accelerate the correspondence search by containing orders of magnitude fewer points than there are features in the images [28]. The most popular correspondence search algorithm is 2D-to-3D matching that directly uses the 2D descriptors as the query features to search for the corresponding 3D scene features based on the approximate nearest neighbor. This is followed by the use of Lowe’s ratio test [29] to eliminate the ambiguous matches. However, the Lowe’s ratio test tends to reject more and more correct matches as too ambiguous for larger scenes since the descriptor space defined by the 3D points becomes denser [13]. Therefore, the 3D-to-2D approach, which inversely matches the 3D points in the model against the 2D features in the image, is adopted to register images. The ratio test of the 3D-to-2D algorithm is not sensitive to large scenes as the descriptor space remains relatively constant and is not negatively affected by the density of the 3D model. The efficiency is affected however, when the scenes become larger.

At the core of correct SfM-based localization is the robust estimation of accurate 2D-3D matches. Due to large viewpoint changes and repetitive textures, using the above-mentioned correspondence search algorithm alone may fail to register an image affected by a high outlier ratio. In order to improve the recall rate, Svarm et al. [30] and Zeisl et al. [27] exploited gravity direction and the height of the camera from inertial measurement unit (IMU) measurements as prior information to create a probabilistic model for the camera pose estimation, which is able to handle an inlier ratio of 1% or less. However, this method requires accurate estimation of the height and gravity direction. Li et al. [31], on the other hand, combined 2D-to-3D matching and 3D-to-2D matching to increase the robustness to high outlier ratios. Mismatches are removed and the lost matches are detected by searching from both side. Sattler et al. [13] adopted the same strategy and extended it to larger scenes by incorporating a bag-of-visual-words model to encode the features. The visibility information embedded in the SfM model was also explored to improve the correspondence search efficiency by getting rid of unrelated images. These methods however, only yield a discrete localization result and apply mainly to outdoor scenes.

Image-based localization in indoor space applications for mobile devices faces challenges. The mobile device is memory and computationally limited, posing a challenge for its practical use in larger scenes, especially in the SLAM mode. Furthermore, changing environments, such as the appearance aliasing caused by illumination or structural change, demand a method that can robustly recognize the right place. Research has explored potential solutions to these problems; FAB-MAP [32], fast appearance-based mapping, is a typical approach for obtaining a location from a single image, based on bag-of-words image retrieval. This solution is widely used in online loop closure detection in mobile robotics, as the algorithm is scalable and adapts in a linear fashion to changes in the number of points and the size of map. In contrast, rather than using a single image, SeqSLAM [33] selected the best candidate location within an image sequence, improving the robustness to extreme environmental change such as moving from daytime to nighttime, from season to season, or from fair weather to rain. Similar to the SeqSLAM method, which uses the image sequence, ABLE-M [34] deploys a binary description of images that reduces memory and computational costs, remaining stable despite environmental changes that affect image appearance. Nowicki et al. [35] evaluated the application of single image and image sequence localization in indoor environments on mobile devices, validating the feasibility and real-time performance of both of these algorithms. Furthermore, Nowicki et al. also found that algorithms using single images are not susceptible to local self-similarity issues inside buildings, as texture changes in images are not as large as those images taken in outdoor space. Thus, we infer that an image sequence is more suitable in situations with a known trajectory but does not perform well at junctions or in open-spaces. Consequently, based on the existing research, we adopted the single image strategy, as it conforms the typical patterns of human movements through space and scalable to larger scenes in indoor localization. Although the single image strategy only obtains a user location for discrete set of positions and is not applicable to highly occluded spaces; nevertheless, this method can be combined with other indoor localization technologies, such as PDR to achieve continuous localization. Moreover, PRD results can constrain image-based localization to the proper locations, avoiding false recognition of places with an appearance similar to the target location.

Based on the above observations, we propose a simple but efficient indoor localization approach that combines image-based localization and PDR for long-term indoor pedestrian trajectory estimation. Considering the memory and computational limitation of the currently available smart phones, we reconstruct the 3D scene model of the indoor environment with the SfM pipeline in advance. In the localization stage, we take an image with the smartphone and match it against the database through a fused 2D-to-3D and 3D-to-2D matching scheme. The image-based localization acts as the starting position for PDR and provides regular correction once the accumulative error is beyond the predefined threshold. We make three main contributions. Firstly, we perform the 3D-to-2D matching only when the 2D-to-3D matching cannot successfully register an image, to achieve a higher image recall rate while restraining the computation time. Secondly, we adopt the visibility and co-visibility information readily encoded in the SfM pipeline in searching for correspondences in both sides, by eliminating irrelevant images and facilitating robust matching. As for the third major contribution, we demonstrate that combining image-based localization and PDR can serve as a promising, low-cost, self-dependent and continuous indoor pedestrian localization strategy, especially in the situations where no map constraints can be exploited. From the experimental results, the proposed method offers an accurate and continuous trajectory estimation, with an error of only 0.56 m, based totally on the smartphone.

## 2. Materials and Methods

**Figure 1:**
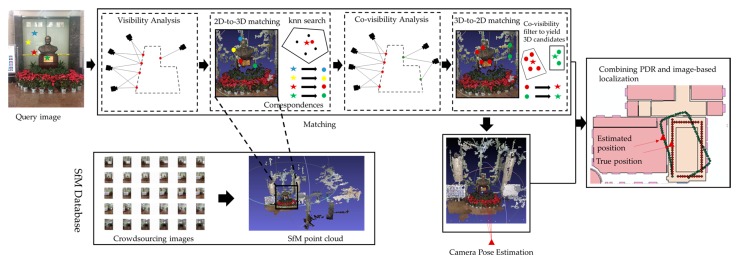
The workflow of the image localization based pedestrian trajectory estimation algorithm.

In this section, we describe our combined image-based localization and PDR framework, which allows for fast and accurate indoor pedestrian localization. The workflow of the proposed localization algorithm is illustrated in Figure 1. We propose a simple but efficient 2D-to-3D and 3D-to-2D combination algorithm to estimate robustly the correspondences, with the 3D-to-2D matching triggered only when not enough valid matches are detected. In order to improve the search efficiency from both sides, the visibility and co-visibility information is adopted to remove the points that are less likely to generate potential matches. An early termination approach is also performed to stop the search once $N_{t}$ matches are found. When combined with the PDR trajectory, we can obtain a bias-free estimation of the pedestrian locations.

### 2.1. Database Construction

Considering the memory and computational constraints of smartphones, in contrast to the SLAM algorithm, we adopt the strategy of reconstructing the 3D model of the indoor scene in advance and perform image-based localization only when lost. The constructed database consists of 3D points with descriptors, using image matching and the VisualSfM (VSFM) algorithm [15]. Since each point of the SfM model is triangulated from the feature traces of multiple images, it is often associated with a cluster of related feature descriptors. Storing all the feature descriptors of each 3D point is straightforward and offers the most accurate description of the local appearance of a point. However, it also induces a high memory requirement and restrains the computational efficiency as some points may have hundreds of associated descriptors [28]. A more concise alternative is to store a single descriptor for each point. We use the mean of the corresponding image feature descriptors to represent each point and a typical way of storing descriptors in a memory-limited environment. While this may not necessarily be representative for clusters of scale-invariant feature transform (SIFT) features that are large, Li et al. [31] proved that this approach can achieve a comparable accuracy to the all-descriptors representation. Other concise descriptor representations such as the median of a related descriptor was also explored but these failed to achieve a performance comparable to the mean SIFT descriptors [28].

### 2.2. Image Registration

In order to obtain immediate feedback after taking a photo of the indoor scene, the ultimate goal of our system is to produce an accurate and efficient pose estimation of a query image, given a relevant database of recovered 3D points. At the core of this is the accurate and efficient 2D-3D correspondence search, whose common pipeline can be described as: for each query feature f with descriptor $d_{f}$, we search the two nearest neighbor points $p_{1}$ and $p_{2}$ with descriptor $d_{p_{1}}$ and $d_{p_{2}}$, by calculating the Euclidean distance between the descriptors and applying the kd-tree searching algorithm. In order to eliminate the ambiguity caused by repetitive textures, the matched correspondences are accepted as valid only when the Lowe’s ratio test is passed:

(1) $${\left\|d_{f}-d_{p_{1}}\right\|}_{2}<\tau \cdot {\left\|d_{f}-d_{p_{2}}\right\|}_{2}$$

where τ is a parameter that is experimentally ranged between [0.6, 0.8] [12]. The generated set of 2D-3D matches are finally input to the RANSAC [36]-based 6-point algorithm, which estimates the pose of the camera [37] by iteratively calculating the transform matrix and choosing the best one with the most inliers. In our method, we consider a query image as successfully localized if the best pose found by RANSAC has at least 12 inliers. The algorithm can be summarized as Algorithm 1.
**Algorithm 1.** 2D-to-3D matching**Input:** Query image feature set $F=\{f_{1},f_{2},\ldots ,f_{n}\}$, with descriptor $D_{f}=\{d_{f1},d_{f2},\ldots ,d_{fn}\}$, and 3D point database $P=\{p_{1},p_{2},\ldots ,p_{m}\}$, with descriptor $D_{p}=\{d_{p1},d_{p2},\ldots ,d_{pm}\}$.**for**
*i* = 1 … n (n is the number of features in an image) Search two nearest neighbor points $p_{j}$ and $p_{h}$ in P for each query feature $f_{i}$, by calculating the nearest distance of the associated descriptor $d_{pj},d_{ph}$ and descriptor $d_{fi}$ using the FLANN algorithm. Perform Lowe’s test, if ${\left\|d_{fi}-d_{pj}\right\|}_{2}<\tau \cdot {\left\|d_{fi}-d_{ph}\right\|}_{2}$, then $\left\{f_{i},p_{j}\right\}$ is regarded as a valid match.**end** Calculate the camera pose by performing 6-point algorithm based on the 2D-3D matches $\left\{f_{i},p_{j}\right\}$.**Output:** a 6-degree camera pose.

However, following the typical workflow as described in this section, the query image may not be registered successfully all the time, especially in the case of large outlier matches being detected. The high outlier rate is caused by either the mismatches of the SIFT descriptors or the high true negative rate of Lowe’s ratio test in the 2D-to-3D matching, especially when the scene gets larger. This is because the descriptor space defined by the 3D points becomes denser for a larger scene, making the Lowe’s ratio test reject more and more correct matches as too ambiguous. As a compromise to the high outlier ratio, RANSAC requires more iteration to ensure the accuracy of the estimated pose, which negatively affects the efficiency of the algorithm. Therefore, other algorithms must be explored for robust estimation of 2D-3D matches.

3D-to-2D matching, which inversely matches 3D points against 2D features, is proposed to recover the lost matches rejected by the 2D-to-3D matching in Lowe’s ratio test, as described in Algorithm 2. The density of the descriptor space defined by the query image does not depend on the model and thus not affected by the scale of the scene. However, it tends to accept false matches because there is no global constraint on the 3D points. When considering the 3D points independently, the Lowe’s ratio test is likely to accept matches for all the 3D points in the set if one of the points passes the test, leading to a significantly higher false positive matching rate. On the other hand, if we suppose that P represents all the 3D points in the model and F represents all the features in the query image, then the time complexity for 2D-to-3D matching is O(|F|log|P|) and the 3D-to-2D matching is O(|P|log|F|). It is easily observable that even in a compact scene, the number of points is much larger than that of detected query features in the image. In other words, 3D-to-2D matching is not as efficient as 2D-to-3D matching when a large scene with extensive 3D points is considered. As reported in [13], when compared to other algorithms, 3D-to-2D matching does not perform well in considering the number of localized images. Therefore, in order to exploit the merits of both methods, a combination must be exploited and a more compact 3D scene must be constructed to accelerate the matching process.

**Algorithm 2.** 3D-to-2D matching**Input:** Query image feature set $F=\{f_{1},f_{2},\ldots ,f_{n}\}$, with descriptor $D_{f}=\{d_{f1},d_{f2},\ldots ,d_{fn}\}$,and database points $P=\{p_{1},p_{2},\ldots ,p_{m}\}$, with descriptor $D_{p}=\{d_{p1},d_{p2},\ldots ,d_{pm}\}$
**for**
*i* = 1 … m (m is the number of 3D points in the database) Search two nearest neighbor features $f_{j}$ and $f_{h}$ from feature set F for the query point $p_{i}$, by calculating the distance between associated descriptor $d_{fj},d_{fh}$ with descriptor $d_{pi}$, using the FLANN algorithm; Perform Lowe’s test, if ${\left\|d_{pi}-d_{fj}\right\|}_{2}<\tau \cdot {\left\|d_{pi}-d_{fh}\right\|}_{2}$, then $\left\{p_{i},f_{j}\right\}$ is regarded as a valid match.**end** Calculate the camera pose by performing a 6-point algorithm based on the 3D-2D matches $\left\{p_{i},f_{j}\right\}$.**Output:** a 6-degree camera pose.

### 2.3. Visibility and Co-Visibility Information

Searching for correspondence from both sides inevitably increases the time needed to detect matches. In order to counteract the time consumption of the extra search process and improve the robustness of the search algorithm, we exploit the visibility and co-visibility information that encodes the underlying geometry in the SfM model, as well as an early termination strategy, to facilitate the correspondence search.

**Figure 2:**
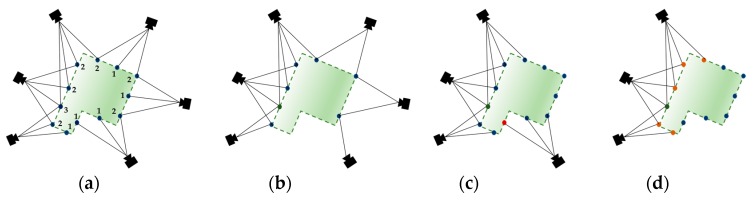
Visibility and co-visibility information. (**a**) Visibility graph. The original graph encodes camera and its corresponding visible points from the process of SfM pipeline. The blue points represent the 3D space, the black line represents the ray linking camera center and the points. The number indicates the number of cameras that observe the point. (**b**) The graph after applying the visibility filter with N=1. (**c**) Co-visibility does not necessarily imply spatial continuity, i.e. the green point and red point are spatially close to each other but are not co-visible. (**d**) This figure shows the graph after applying the co-visibility filter for the green points. The orange points depict all the co-visible points (red points) of the green points; blue points are not co-visible.

Visibility information is rooted in one of the major properties of SfM, in that each 3D point is recovered from several image features, which define the visibility significance of the point. The points with higher visibility have a larger possibility of being matched than the points with lower visibility, for the reason that a highly visible point is intuitively more likely to be visible in the query image [31]. In the 2D-to-3D search, the image features search through the whole database and find a corresponding 3D point in a limited space, making large search work wasted. This implies that the correspondence search could be accelerated if we could generalize the 3D model with the points that are more likely to yield a match. We therefore apply a visibility filter to the SfM model and preserve a set of points that are visible in more than N images (N = 5 in our experiment) as a simplified model (as shown in Figure 2a,b). The correspondence search is then performed between the query image and the simplified model until $N_{t}$ matches are found. $N_{t}$ controls the balance between run-time efficiency and localization effectiveness.

The search algorithm can be formulated as follows. Firstly, we determine the subset $P^{\prime }=\{P^{\prime }\in P|P_{i}>0\}$ that contains all the potential points that survive in the visibility filter. We then search for the 2D features against the 3D point subsets, such that the number of expected matches is at least $N_{t}$. If the algorithm finds $N_{t}$ matches successfully, then the set of matches $M=\{(f,p)|f\in F,p\in P^{\prime }\}$ links the 2D features in the query image directly to the 3D points in the model. These matches are fed directly into the pose estimation routine. We use the 6-point direct linear transformation (DLT) approach to solve the projection matrix of the query camera, followed by local bundle adjustment to refine the pose.

However, there may exist situations where not enough valid matches can be found, due to the uneven distribution caused by preserving only the highly visible points. We therefore perform the 3D-to-2D matching to recover the lost matches. This begins with the 3D point set p obtained by performing the 2D-to-3D matching. We then apply k-nearest neighbor search to generate the set $p_{n}$, resulting in a set of potential valid 3D-to-2D correspondences. However, the spatial proximity does not necessarily imply the matching correspondence. For example, in Figure 2c, the spatially close red and green points can never be observable in the same query image. Therefore, we exploit the co-visibility filter to remove such confusing neighbor points.

As illustrated in Figure 2, co-visibility can be defined using a bipartite visibility graph. Each point refers to a 3D point in the model and each node refers to a camera. The edge $e=\{p_{G},c_{G}\}$ connects the 3D points and camera. The camera sets that observe the same points are thus defined as:

(2) $$C_{G}(p)=\{c_{G}\in C_{G}|\{p_{G},c_{G}\}\in E\}$$

where $p_{G}$ represents the 3D points and $c_{G}$ represents the cameras. Consequently, the co-visible point sets G(M) contain the largest component that is observed by camera sets $C_{G}(p)$ (as shown in Figure 2d). The points that are spatially continuous but do not imply co-visibility may be confusing and contaminate the generation of correct correspondences. Therefore, through the co-visibility filter, we remove the points that are not contained in the bipartite graph, which means that the whole search set is a subgraph consisting of only the matching points and their cameras. Instead of applying RANSAC-based pose estimation on all the matches, our co-visibility filter thus first identifies all the connected components and then filters out all the matches not contained in G(M). By eliminating the wrong matches, the RANSAC-based pose estimation is accelerated and has a larger possibility of obtaining correct answers. The proposed algorithm is presented as Algorithm 3.

**Algorithm 3.** The proposed 2D-to-3D/3D-to-2D matching**Input:** Query image feature set $F=\{f_{1},f_{2},\ldots ,f_{n}\}$, with descriptor $D_{f}=\{d_{f1},d_{f2},\ldots ,d_{fn}\}$, and database points $P=\{p_{1},p_{2},\ldots ,p_{m}\}$, with descriptor $D_{p}=\{d_{p1},d_{p2},\ldots ,d_{pn}\}$ Preserve the points that are visible in more than five images: $P_{v}=VisibilityFilter(P)$, $P_{v}=\{p_{1},p_{2},\ldots ,p_{x}\}(x\leq n)$.**for**
*i* = 1 … x Perform 2D-to-3D algorithm with early termination: $\left[F_{v}^{\prime },P_{v}^{\prime }]=2Dto3Dmatch(F,P_{v}\right)$.**end****If** inlier > threshold**end****else** Eliminate non-covisible points: $P_{cv}=CoVisibilityFilter(P_{v}^{\prime })$, $P_{cv}=\{p_{1},p_{2},\ldots ,p_{y}\}(y\leq x)$. **for**
*j* = 1 … y Perform 3D-to-2D algorithm: $\left[P_{cv}^{\prime },F_{cv}^{\prime }]=3Dto2Dmatch(P_{cv},F_{cv}\right)$. **end****end** Calculate the camera pose: $C=RansacCameraPoseEstimation(F_{v}^{\prime },P_{v}^{\prime })$.**Output:** a 6-degree camera pose.

### 2.4. PDR Combination

The image-based localization can obtain accurate pose estimation in discrete places. However, in most cases, we need continuous tracking of the pedestrian in applications such as indoor navigation and augmented reality. The PDR algorithm, which estimates the distance and heading measurements of every step, given an initial location from the accelerometer and gyroscope embedded in the smartphone, has become a promising low-cost and continuous localization technology in indoor environments. Four core components are considered in PDR: step detection, step length estimation, heading estimation and initial position determination.

The step detection algorithm relies on the fact that the accelerometer reveals a repetitive pattern when the user walks. We use a two-threshold based peak detection algorithm to identify the peaks. The first threshold is the minimum acceleration magnitude that determines a peak and the second threshold is the minimum time duration between two steps. Peaks that satisfy both the magnitude and frequency threshold are identified as true steps. The orientation is estimated from the gyroscope by exploiting the quaternion calculation. Our system assumes that the smartphone is held in hand, with it pointing in a forward direction. The angular rate reading from the gyroscope is then integrally calculated to determine the orientation quaternion at each step. The orientation quaternion (or the Euler rotation vector roll-pitch-yaw (φ,θ,ψ) between two successive epochs and a scalar component) from the gyroscope is then utilized to approximate the orientation update. The step length is calculated from the Weinberg model [17]:

(3) $$length=K\cdot \sqrt[{4}]{a_{\max}-a_{\min}}$$

where $a_{\max}$ and $a_{\min}$ are the maximum and minimum values of the yaw acceleration samples, respectively. K is a constant determined by training. Our approach takes K=0.46 as the initial value. Since step length exhibits variation, even with the same individual in the same walk, we add a random error δ (uniformly distributed in the range of ±10%) to the stride length.

However, due to the low-cost nature of the micro-electromechanical system (MEMS) sensors, the long-term tracking of PDR may locate the user several meters away from the true location. Moreover, the initial location must be provided by other absolute positioning technologies, which means that the PDR method alone is unable to achieve acceptable results. To solve this problem, we combine image-based localization with PDR to correct the biased trajectory and provide reliable long-term indoor localization.

**Figure 3:**
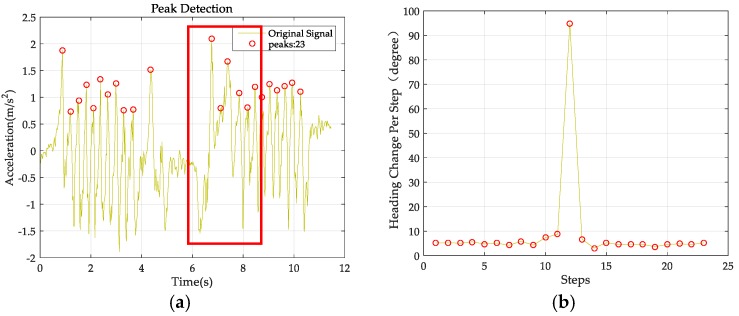
Photo detection. (**a**) The accelerometer reading when taking a photo; (**b**) Heading estimation from the gyroscope when taking a photo.

The image-based localization provides the initial location and regular correction for the PDR when lost. After obtaining the image-based localization result and the PDR trajectory, we now combine them. The key problem is identifying the action of taking a photograph and discriminating it from the PDR step counting procedure. Therefore, we analyzed the pattern of the accelerometer and gyroscope when taking a photo with the smartphone. The user was asked to walk a distance as usual, with the smartphone held in hand. After a while, the user was asked to stop to take a photo of the environment and then continue to walk. The readings of the accelerometer and gyroscope are shown in Figure 3.

The action of taking a photograph reflects on the accelerometer as a slight wave between two steps and on the gyroscope as a sharp increase and then back to normal (as shown in Figure 3). The pattern reflected by the accelerometer may be confused with the action of standing still. In addition, the two starting and ending peaks detected by the accelerometer are not real steps and may contaminate the step count because the sensor reading of the user raising the phone and then moving it back to its original position is similar to that of regular walking. This can be discriminated from a standing still action by detecting the sharp orientation change on the gyroscope reading. Any of the single readings in the xyz coordinates of the orientation may result in a different pattern due to the jitter of the smartphone. Therefore, we use the mean-square-root of the three axes for detection, which show a large, sharp change in orientation. The combinational detection of the accelerometer and gyroscope can discriminate the action of taking a photograph from both staying still and walking.

**Figure 4:**
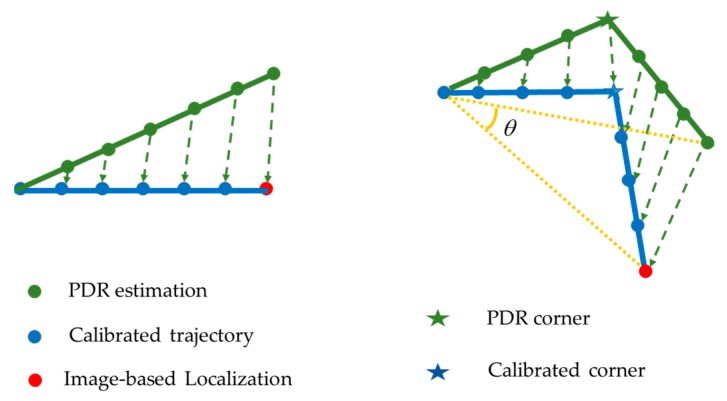
Trajectory back-tracking estimation.

The PDR stops step counting when detecting the action of taking a photograph and resumes working after receiving the image-based localization result and treating it as the initial location of the current position estimation. Because of the trajectory drift caused by accumulative error, there may be a discrepancy between the newly relocated result and the last location estimated by PDR, indicating that large errors exist in the PDR-estimated trajectory. In order to reduce this discrepancy, we inversely recalibrate the PDR result based on the image-based localization. Since the errors of the PDR are accumulated step-by-step, we backward counteract the error linearly as the number of steps. If the past trajectory is a straight line, the linear transformation is performed directly. If the past trajectory consists of several lines with corners, we first perform rotation transformation according to the corner of two lines connecting the start point and the two endpoints and then segment the trajectory into several parts with corners. Finally, we apply a linear transformation to each segment as in line transformation. The process is illustrated in Figure 4. However, even the estimated trajectory may not perfectly reflect the trajectory of the pedestrian. For example, the user is supposed to have made an orthogonal turn but the estimated orientation is larger than 90°, as shown in Figure 4. However, this minor error has no significant negative impact on the estimated localization. It is within the tolerance error of indoor pedestrian localization and does not require absolute accuracy. It is an image-based localization, which avoids large location mistakes and PDR continuously tracks the pedestrian trajectory that jointly determines the effectiveness of the localization result. In this way, we achieve continuous and accurate pedestrian trajectory estimation in an indoor environment.

## 3. Experiments

In order to verify the proposed image localization aided pedestrian trajectory estimation algorithm in indoor scenes, several experiments were conducted to localize pedestrians using smartphones in the State Key Laboratory of Information Engineering in Surveying, Mapping and Remote Sensing (LIESMARS) building of Wuhan University. In this section, we first evaluate the efficiency and effectiveness of the proposed image-based localization algorithm. The PDR trajectory is then shown to analyze how the error accumulated. Finally, we apply the image-based localization result to the PDR algorithm and investigate how they can be appropriately combined.

### 3.1. Image-Based Localization

#### 3.3.1. Database Construction

**Figure 5:**
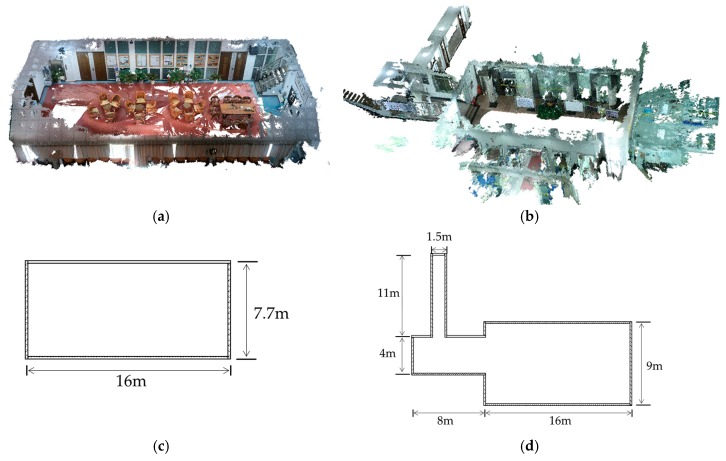
The dense 3D models of the reconstructed datasets. (**a**) A model of the meeting room dataset; (**b**) A model of the lobby dataset; (**c**) The floor plan of the meeting room dataset; (**d**) The floorplan of the lobby dataset.

Two indoor scenes of the LIESMARS building were reconstructed by VSFM from the crowdsourced images taken by three volunteers with an iPhone 7 (Apple, Cupertino, CA, USA), an iPhone 7 Plus smartphone and a Cannon EOS 6D SLR (Tokyo, Japan), respectively. The Meeting Room scene has an area of 16×7.7 m, with 261 images taken. The Lobby scene has an area of about 24×18.5 m, with 343 images taken. The details of both scenes are shown in Figure 5. The database for both scenes appears in Table 1. There is no standard regulation showing how many images are needed to obtain a sufficient environment model. However, coverage of over 60% between adjacent images is suggested when collecting data. Furthermore, images at higher resolution tend to reconstruct a more qualified model. This is because the SfM models are triangulated from features detected in the image, a higher image resolution means more densely and accurately detected features. As for the topologies of the environment, more images between the junctions are required to maintain visual connection between the structures; as no obvious difference will exist in the structures (for example the corridors, loops and open areas) once coverage of over 60% between adjacent images is satisfied. Specifically, the Lobby scene contains three different structures including a corridor, a room and open space, captured in 13, 41 and 289 images, respectively.

For each dataset, only the 3D points that were visible in more than two cameras were kept in the model. The query images were additionally taken to evaluate the image localization algorithm and were not used in the database construction. For the points with many descriptors, we calculated the mean descriptors and used them for the feature matching. This can reduce the memory requirement and improve the efficiency, without loss of accuracy, when compared with the all-descriptors strategy. To obtain the real scale of the indoor model, we put four markers with known lengths in the indoor scenes and made sure that each of them could be seen in at least four images to recover the real scale of the model. The calibrated models were then transformed into the predefined indoor coordinates using SfM_georef [38]. Therefore, the reconstructed poses can be treated as the ground truth, with the distances measured in meters.

#### 3.3.2. Image Pose Estimation

After obtaining the database of the indoor scenes, we then estimated the pose of the query images using the standard 6-point DLT (p6p) algorithm, which computes the projection matrix from six 2D-3D matches [37] inside a RANSAC loop. The Lowe’s test $L_{t}=0.7$ was applied for the 2D-to-3D matching and $L_{t}=0.6$ for the 3D-to-2D matching. A query image is considered successfully localized if the best pose found by RANSAC had at least 12 inliers. The correspondence searching was accelerated by employing the kd-tree, visiting 10 leaves to compute the assignments, using a modified implementation from the Fast Library for Approximate Nearest Neighbors (FLANN) library [39]. The pose estimation was conducted on a Lenovo ThinkPad X240 laptop (Beijing, China), where the database was stored. When a query image was taken with the smartphone, it was transferred to the laptop via the Internet (through Wi-Fi) for pose estimation. The result was then returned to the smartphone for later use.

**Figure 6:**
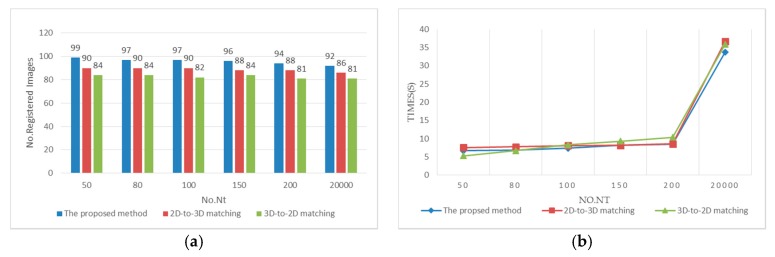
Comparison of the three algorithms with parameter $N_{t}$: (**a**) comparison of the effectiveness of the three algorithms; (**b**) comparison of the efficiency of the three algorithms.

We report and compare the localization results obtained on the two datasets using three algorithms: 2D-to-3D matching, 3D-to-2D matching and the proposed method. The effectiveness and efficiency of the above algorithms with different values of parameter $N_{t}$ (the maximum matched correspondences) is shown in Figure 6. The effectiveness is measured with the number of localized images, while the efficiency is measured with the mean time required to localize an image. As can be seen in Figure 6, $N_{t}=50$ offers the most registered images, as well as the least localization time for all the algorithms. $N_{t}$ controls the termination of the correspondence search after finding $N_{t}$ matches, avoiding unnecessary matching of all the query features. With the increase of $N_{t}$, the number of registered images is not increased but reduced. The reason for this may be that indoor scenes with fewer 3D points have a sparser descriptor space, resulting in a higher false positive matching rate. The time complexity for the combined 2D-to-3D and 3D-to-2D matching is O(|F|log|P|+|P|log|F|). Actually, the visibility filter and co-visibility filter largely reduce the points involved in computation, especially when the scene gets larger. If the density of the points is evenly distributed across the space, the visibility filter uniformly eliminates the points participating in computation. On the other hand, the co-visibility filter retains points only near the targeting points, eliminating large proportions of unrelated points, which makes the search-time increase logarithmically with the size the model. As more feature correspondences are used for the matching, the higher mismatches make the inlier ratio lower than the threshold ratio R=0.2, which therefore is considered as a registration failure and reduces the number of registered images.

The proposed method exhibits better localization effectiveness than the other methods because it applies 3D-to-2D matching if the pose cannot be estimated from 2D-to-3D matches alone. In this way, the lost matches can be recovered and thus increase the number of registered images. However, the proposed method cannot achieve the same efficiency with 3D-to-2D matching when the maximum matches are few in number. This is due to the correspondence search from two sides requiring extra computation. However, as the maximum match number increases, the proposed method can achieve a comparable efficiency to the other methods by executing a visibility filter to construct a simplified model for the 2D-to-3D matching and a co-visibility filter to remove 3D points unlikely to yield matches from the candidate list for 3D-to-2D search. In this way, the localization time is reduced after eliminating potentially wrong 2D-3D correspondences before applying the RANSAC-based camera pose estimation. With the proposed image-based localization algorithm, we obtained a mean localization error of 0.50 m in the lobby dataset and 0.36 m in the meeting room dataset. The mean localization error was calculated as the distance between the estimated camera center and the ground truth position for the camera. The ground truth is the position where the person stands and takes the photo and is not exactly the position where the smartphone lies.

### 3.2. PDR Trajectory

In this section, we evaluate the effectiveness of localizing pedestrians using PDR alone. The volunteers were asked to walk along a predefined route holding the smartphone in hand and the accelerometer and gyroscope readings were simultaneously exploited for PDR estimation. Given the initial position, the relative movement can be estimated from the step detection, step length estimation and heading estimation. All three modules may induce error but the error of the heading estimation has a dominant impact on the eventual result. We evaluated the most difficult situation by asking the volunteers to walk with many orientation changes. The experimental route was a rectangular route, without additional constraints from maps to help estimate the results. A Xiaomi 2 (Beijing, China) smartphone with Android (Google, Mountain View, CA, USA) operating system was used in the experiment.

**Figure 7:**
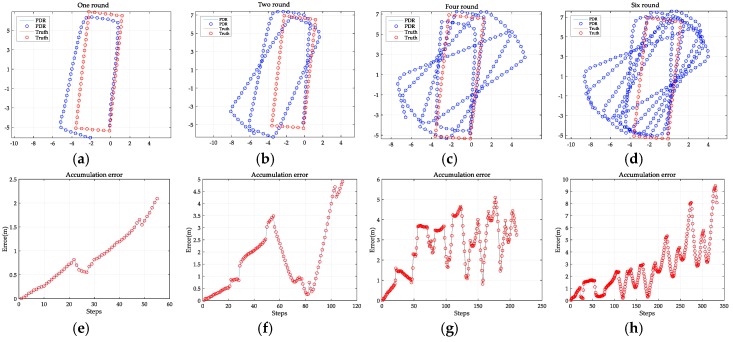
The trajectory and accumulative error of PDR: (**a**–**d**) are the estimated trajectories of walking around the rectangular route from circuit one to circuit six; (**e**–**h**) are the corresponding accumulative errors of (**a**–**d**).

Table 2 reports the step detection result of the PDR algorithm, which achieves a mean accuracy of 98.71%. The estimated trajectory and the accumulative errors are illustrated in Figure 7. It can be seen that PDR can accurately locate the users in short-term tracking but it fails to locate them accurately in extended routes. Specifically, 330 steps of walking a complex route (containing multiple turns) allows us to locate the user with a mean error of 2.66 m and a maximum error of 9.44 m. The results therefore demonstrate that PDR alone cannot provide reliable indoor localization.

### 3.3. Image Localization Based Trajectory Estimation

The experimental results obtained with the PDR algorithm indicate that PDR can lead to a large accumulated error in long-term tracking. However, the short-term routes often exhibit a satisfactory result. We therefore partition the long trajectories into several short ones and aim to achieve accurate localization in each sub-trajectory. The image localization result can serve as the partitioning point and provide the initial localization and orientation. Two kinds of partitioning strategies can be exploited. The first is to use constant time to resume a PDR and the second is to use constant step. Since situations exist where the user stands or sits still so that no error is accumulated, we prefer to relocate the user by the step frequency.

**Figure 8:**
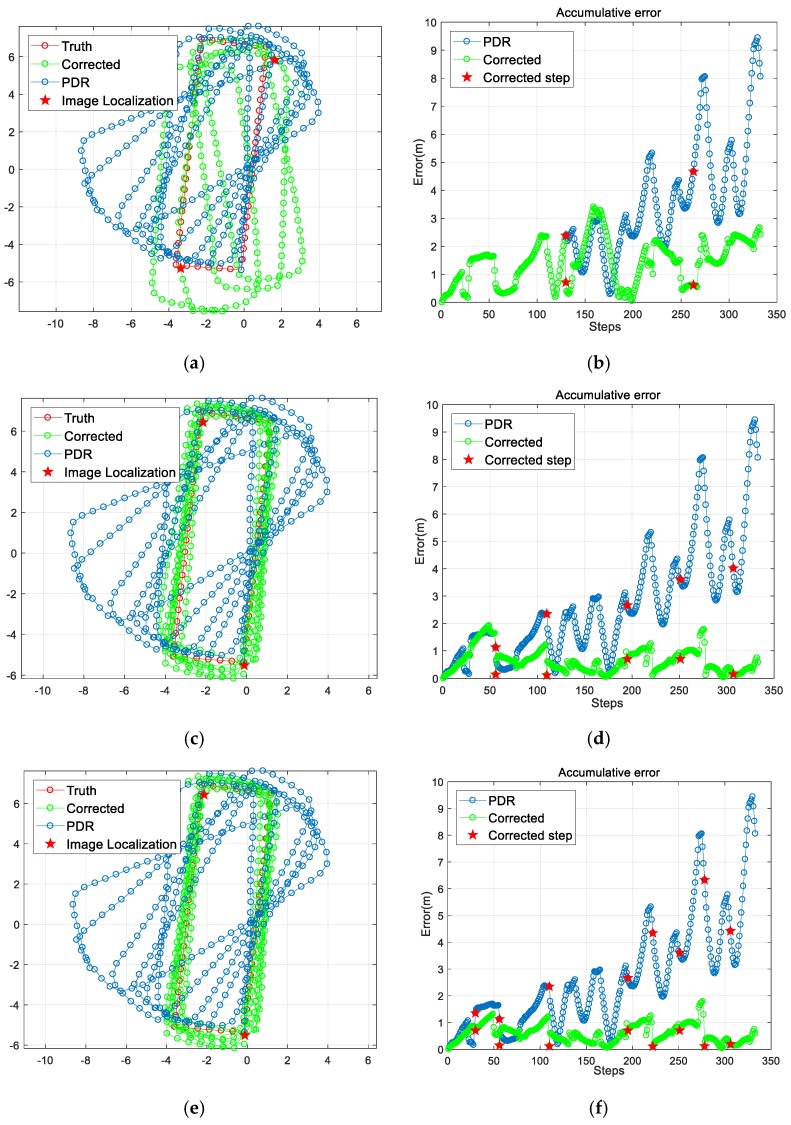
The results of the image localization aided pedestrian trajectory estimation: (**a**,**c**,**e**) are the estimated results when correcting the trajectory every 130 steps, every 55 steps and every 28 steps, respectively; (**b**,**d**,**f**) are the corresponding accumulative errors of (**a**,**c**,**e**).

The accumulative error shows a non-monotonic increasing trend as the user walks a circular route. We set the step threshold to be 130, 55 and 28, respectively and we resumed the PDR estimation once the step threshold was reached. Table 3 and Figure 8 detail the estimated trajectories and the accumulative error. As shown, the more frequent the use of image-based localization to correct the trajectory, the higher the accuracy of the estimated result. The dominant source of error is deviation in the heading estimation, causing trajectory bias away from the truth. However, with frequent correction from the image-based localization result, the pedestrian estimation can achieve a mean accuracy of 0.56 m, with a maximum error of 1.78 m.

**Figure 9:**
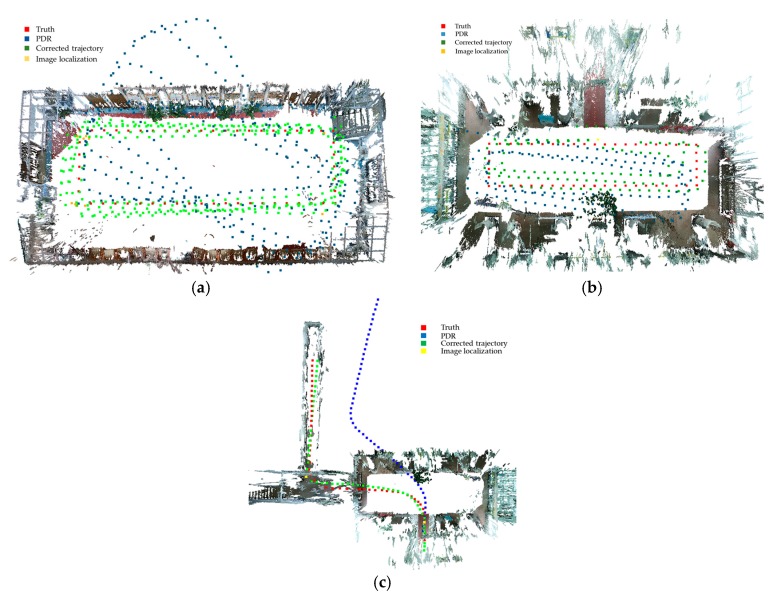
The localization results shown in the indoor environment: (**a**) The localization result for the meeting room dataset; (**b**) The localization result for the lobby dataset; (**c**) The localization result for the level-scale dataset in the lobby dataset.

Figure 9 presents the estimated trajectories through the combination of image-based localization and PDR in the SfM-reconstructed indoor point cloud model. The level-scale trajectory was estimated to demonstrate the efficiency and accuracy of the proposed approach (shown in Figure 9c). The user was asked to walk through the open area, turn left into the small room and turn right to the corridor. In the process, the user performed two image-based localization actions. The first time when the user walked in the open space and second when the user walked into the corridor. It takes about 7.65 s to localize an image when terminating correspondence search when 100 matches were found, a slight increase when compared with localizing in a single room. Furthermore, the localization accuracy was largely improved when applying the image-based localization. In the case of a multi-floor scene, the user location can be used as a prior to constrain the correspondence search to a certain floor, keeping the time complexity within the acceptable range. The furniture was removed to show the trajectories clearly. This realistic model with a specific indoor structure shows clearly that the proposed image-based localization aided pedestrian tracking algorithm can effectively reduce the accumulative error in PDR and the estimated trajectory converges with the truth. The pedestrian can be continuously tracked in the indoor space and we can avoid the phenomenon of crossing a wall or mistakenly being localized in another room.

## 4. Discussion

### 4.1. The Effectiveness of Image Registration

From the above experiments, we can conclude that the effectiveness of the image-based localization significantly influences the validation of the final localization. The image-based localization can contaminate the final localization in two ways. The first is that the images cannot be registered and the second is that the images are registered but with large errors. In order to guarantee that the image-based localization can obtain a successful result, we evaluated the factors that may lead to an invalid result. Four aspects were evaluated:

- Texture: few or intensive;
- Image perspective: orthogonal or large perspective view;
- Distance: far, median, or near;
- Image pixels: Cannon EOS SLR or smartphone.

**Figure 10:**
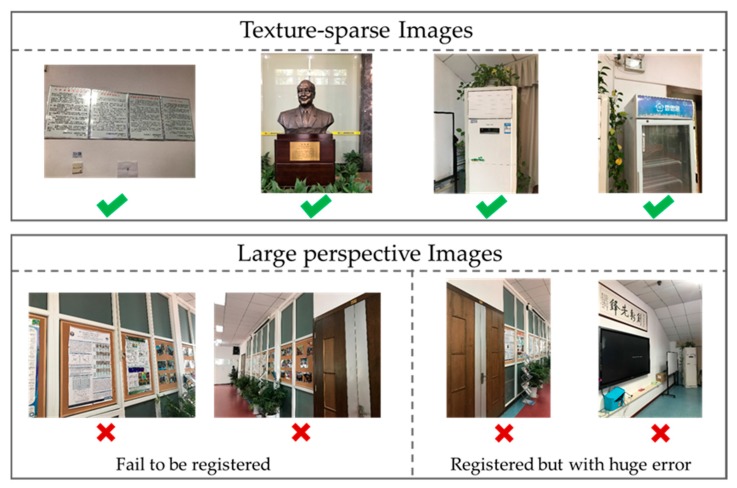
Examples of query images: The images in the first row have sparse textures but were successfully registered. The images in the second row could not be localized for large perspective, the left two pictures were not registered, while the right two pictures were registered but with huge errors.

We assigned each of the factors 20 images for the experiments and calculated the number of registered images. It turns out that distance and image pixels have no obvious influence on the registration effectiveness. In addition, images with few textures (as shown in Figure 10) are, surprisingly, as successful in registration as those images with abundant textures. The worst result comes from images with a large perspective view (as shown in Figure 10). The failure of the registration is caused by either too few inlier matches or a too-small inlier ratio. The registration error is caused mainly by the latter. This can be solved by increasing the inlier ratio threshold that determines the minimum inlier ratio of registration. However, this may also decrease the number of registered images. Therefore, an appropriate way may be to construct synthetic views [24], or to simply eliminate those images with a large perspective from the registration.

### 4.2. The Accuracy of Combined Localization

**Figure 11:**
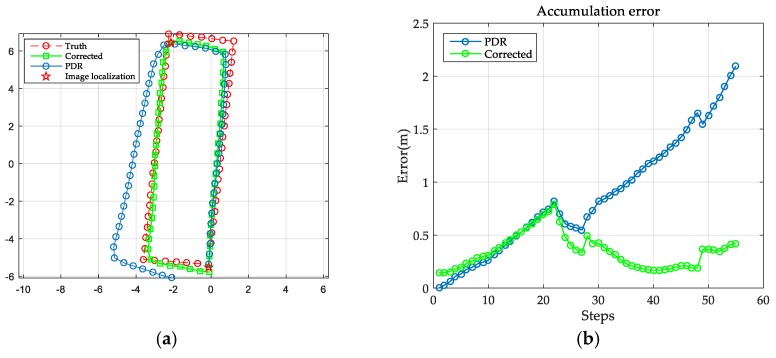
Map-constrained trajectory estimation: (**a**) is the map-constrained result of the trajectory estimation; (**b**) is the accumulative error.

The above experiments show that frequent correction using image-based localization can achieve a sub-meter localization error. However, when performing image-based localization with a large step duration, trajectory bias may still induce localization error. This could be alleviated by introducing map constraints. Map constraints can help predict the behavior pattern of humans; for example, when walking in a corridor, the user tends to follow a straight line. This can be used to counteract the negative influence of the heading estimation. Figure 11 illustrates the map-constrained result of the proposed method when the user walks in a corridor with four turns. The result shows that the error caused by the PDR estimation during the sub-trajectories can be corrected by the map topology. The estimated trajectory conforms with the truth by aligning the orientation to the corridor directions. The accumulative error is accordingly reduced. However, this method does not work in situations such as open areas without structural constraints imposed by maps. As a consequence, in our future work, we will incorporate map constraints into the proposed image localization based pedestrian trajectory estimation, to accommodate all kinds of indoor structure, as well as reduce the number of photos required in map-constrained places.

### 4.3. Privacy Issues

Using the smartphone camera may introduce some privacy problems. To protect user privacy concerning cameras, several potential solutions are proposed. First, the user must authorize the camera before using the application and unauthorize it after closing the application. Second, a separate image folder can be created for saving the images for localization, avoiding illegal access to personal images. Third, the application might require a correct passport before reading the image data. Finally, frequent updating and examination is required to guarantee everything is right in case of emergencies.

## 5. Conclusions

In this paper, we have proposed a low-cost and effective indoor localization framework that combines image-based localization and PDR for robust pedestrian trajectory estimation. Compared with the previous methods, our approach has several advantages. By combining 2D-to-3D and 3D-to-2D searches with visibility and co-visibility information readily available in the SfM model, we obtain a high-accuracy estimation of image pose without loss of time efficiency. By frequently performing image-based localization as the initial location for PDR, the long trajectories with large accumulative errors are segmented into short and accurately estimated sub-trajectories. By exploiting crowdsourced images and the inertial sensors (accelerometer and gyroscope) embedded in smart phones, we can achieve fast, self-dependent, low-cost indoor localization, with an accuracy of about 0.56 m.

In our future work, we will exploit the map information to provide topological, geometrical and semantic constraints for more accurate trajectory estimation, further improving the applicability of the low-cost indoor localization technology. Furthermore, we will explore an improved SfM strategy that can be extended to constructing a 3D indoor model from unordered crowdsourced images, facilitating the accessibility to 3D point cloud databases and hence the image-based localization approach.

## Figures and Tables

**Table 1:** The indoor databases used for the evaluation.

Dataset	#DB Images	#3D Points	#Query Images	#Feature
Meeting room	261	74,200	125	74,200
Lobby	343	74,450	112	74,450

**Table 2:** The step detection result of the PDR algorithm.

	One Circuit	Two Circuits	Four Circuits	Six Circuits
True steps	55	112	218	334
Detected steps	57	112	221	333

**Table 3:** Trajectory with different step thresholds.

	No Correction	130 Corrections	55 Corrections	28 Corrections
Mean error (m)	2.66	1.49	0.83	0.56
Max error (m)	9.44	3.40	2.24	1.78

## References

[1] Wang J., Hu A., Liu C., Li X. (2015). A Floor-map-aided WiFi/pseudo-odometry Integration Algorithm for an Indoor Positioning System. Sensors.

[2] Chen Z., Zou H., Jiang H., Zhu Q., Soh Y.C., Xie L. (2015). Fusion of WiFi, Smartphone Sensors and Landmarks Using the Kalman Filter for Indoor Localization. Sensors.

[3] Zhuang Y., Yang J., Li Y., Qi L., Elsheimy N. (2016). Smartphone-Based Indoor Localization with Bluetooth Low Energy Beacons. Sensors.

[4] Zou H., Chen Z., Jiang H., Xie L., Spanos C. Accurate Indoor Localization and Tracking Using Mobilephone Inertial Sensors, WiFi and iBeacon. Proceedings of the IEEE International Symposium on Inertial Sensors and Systems.

[5] Kim H.S., Choi J.S. Advanced indoor localization using ultrasonic sensor and digital compass. Proceedings of the International Conference on Control, Automation and Systems.

[6] Kempke B., Pannuto P., Dutta P. SurePoint: Exploiting Ultra Wideband Flooding and Diversity to Provide Robust, Scalable, High-Fidelity Indoor Localization: Demo Abstract. Proceedings of the ACM Conference on Embedded Network Sensor Systems CD-ROM.

[7] Pasku V., Angelis A.D., Dionigi M., Angelis G.D., Moschitta A. (2016). A Positioning System Based on Low-Frequency Magnetic Fields. IEEE Trans. Ind. Electron..

[8] Kumar S., Gil S., Katabi D., Rus D. Accurate Indoor Localization with Zero Start-up Cost. Proceedings of the International Conference on Mobile Computing and Networking.

[9] Google Project Tango. https://www.google.com/atap/projecttango/Googleprojecttango.

[10] Ruotsalainen L., Kuusniemi H., Bhuiyan M.Z.H., Chen L., Chen R. (2013). A Two-dimensional Pedestrian Navigation Solution Aided with a Visual Gyroscope and a Visual Odometer. GPS Solut..

[11] Zhao L., Huang S., Dissanayake G. Linear SLAM: A Linear Solution to the Feature-based and Pose Graph SLAM Based on Submap Joining. Proceedings of the IEEE/RSJ International Conference on Intelligent Robots and Systems.

[12] He R., Wang Y., Tao Q., Cai J., Duan L. Efficient Image Retrieval Based Mobile Indoor Localization. Proceedings of the Visual Communications and Image Processing.

[13] Sattler T., Leibe B., Kobbelt L. (2016). Efficient & Effective Prioritized Matching for Large-Scale Image-Based Localization. IEEE Trans. Pattern Anal. Mach. Intell..

[14] Snavely N., Seitz S.M., Szeliski R. (2006). Photo Tourism: Exploring Photo Collections in 3D. ACM Trans. Graph..

[15] Wu C. Towards Linear-Time Incremental Structure from Motion. Proceedings of the 2013 International Conference on 3D Vision.

[16] A.LLC Agisoft PhotoScan User Manual. http://www.agisoft.ru/pscan/help/en/pscan_pro.

[17] Harle R. (2013). A Survey of Indoor Inertial Positioning Systems for Pedestrians. IEEE Commun. Surv. Tutor..

[18] Hays J., Efros A.A. IM2GPS: Estimating Geographic Information from a Single Image. Proceedings of the Computer Vision and Pattern Recognition.

[19] Quelhas P., Monay F., Odobez J.M., Gatica-Perez D., Tuytelaars T. (2007). A Thousand Words in a Scene. IEEE Trans. Pattern Anal. Mach. Intell..

[20] Zamir A.R., Shah M. Accurate Image Localization Based on Google Maps Street View. Proceedings of the European Conference on Computer Vision.

[21] Zhang W., Kosecka J. Image Based Localization in Urban Environments. Proceedings of the International Symposium on 3D Data Processing, Visualization, and Transmission.

[22] Crandall D.J., Backstrom L., Huttenlocher D., Kleinberg J. Mapping the World’s Photos. Proceedings of the International Conference on World Wide Web.

[23] Li X., Wu C., Zach C., Lazebnik S., Frahm J.M. (2008). Modeling and Recognition of Landmark Image Collections Using Iconic Scene Graphs.

[24] Chen D.M., Baatz G., Koser K., Tsai S.S. City-scale Landmark Identification on Mobile Devices. Proceedings of the IEEE Conference on Computer Vision and Pattern Recognition.

[25] Torii A., Sivic J., Okutomi M., Pajdla T. (2015). Visual Place Recognition with Repetitive Structures. IEEE Trans. Pattern Anal. Mach. Intell..

[26] Knopp J., Sivic J., Pajdla T. Avoiding Confusing Features in Place Recognition. Proceedings of the European Conference on Computer Vision.

[27] Svarm L., Enqvist O., Oskarsson M., Kahl F. Accurate Localization and Pose Estimation for Large 3D Models. Proceedings of the Computer Vision and Pattern Recognition.

[28] Li Y., Snavely N., Dan H., Fua P. Worldwide Pose Estimation Using 3D Point Clouds. Proceedings of the European Conference on Computer Vision.

[29] Lowe D.G. (2004). Distinctive Image Features from Scale-invariant Keypoints. Int. J. Comput. Vis..

[30] Zeisl B., Sattler T., Pollefeys M. Camera Pose Voting for Large-Scale Image-Based Localization. Proceedings of the IEEE International Conference on Computer Vision.

[31] Li Y., Snavely N., Huttenlocher D.P. Location Recognition Using Prioritized Feature Matching. Proceedings of the European Conference on Computer Vision.

[32] Cummins M., Newman P. (2008). FAB-MAP: Probabilistic Localization and Mapping in the Space of Appearance.

[33] Milford M.J., Wyeth G.F. SeqSLAM: Visual route-based navigation for sunny summer days and stormy winter nights. Proceedings of the IEEE International Conference on Robotics and Automation.

[34] Arroyo R., Alcantarilla P.F., Bergasa L.M., Romera E. OpenABLE: An open-source toolbox for application in life-long visual localization of autonomous vehicles. Proceedings of the IEEE International Conference on Intelligent Transportation Systems.

[35] Nowicki M., Wietrzykowski J., Skrzypczyński P. Experimental evaluation of visual place recognition algorithms for personal indoor localization. Proceedings of the International Conference on Indoor Positioning and Indoor Navigation.

[36] Fischler M.A., Bolles R.C. (1981). Random Sample Consensus: A Paradigm for Model Fitting with Applications to Image Analysis and Automated Cartography.

[37] Hartley R., Zisserman A. (2000). Multiple View Geometry in Computer Vision.

[38] James M.R. Sfm_georef: Automating Image Measurement of Ground Control Points for SfM-based Projects. Proceedings of the EGU General Assembly Conference.

[39] Muja M. Fast Approximate nearest neighbors with automatic algorithm configuration. Proceedings of the International Conference on Computer Vision Theory and Application.

